# Correlation between sialic acid levels in the synovial fluid and the radiographic severity of knee osteoarthritis

**DOI:** 10.3892/etm.2014.1679

**Published:** 2014-04-14

**Authors:** ZHIGANG CUI, KEMIN LIU, ANQING WANG, SIHAI LIU, FEI WANG, JIANJUN LI

**Affiliations:** 1 Rehabilitation College, Capital Medical University, Beijing 100069, P.R. China; 2 Department of Orthopedics and Rehabilitation, China Rehabilitation Research Center, Beijing Charity Hospital, Beijing 100068, P.R. China

**Keywords:** sialic acid, serum, synovial fluid, severity, osteoarthritis

## Abstract

Osteoarthritis (OA) is associated with the presence of inflammation. Sialic acid (SA), an acetylated derivative of neuraminic acid, is reported to be a useful biomarker of inflammation. The aim of the present study was to investigate the correlation between SA levels in the serum and synovial fluid (SF) and radiographic severity in patients with knee OA. A total of 234 patients with knee OA were recruited for the study, as well as 20 patients that had suffered a knee injury or fracture (without knee OA) and 160 healthy controls. Radiological grading of OA in the knee was conducted according to the Kellgren-Lawrence (KL) grading system. SA levels in the serum and SF were measured using Warren’s thiobarbituric acid assay. The results demonstrated that knee OA patients exhibited significantly elevated levels of serum SA when compared with the healthy controls, and also significantly elevated levels of SF SA when compared with the knee fracture patients. Higher SA levels in the SF were identified in knee OA patients with KL grade 4 as compared with patients with KL grade 2 or 3. In addition, OA patients of KL grade 3 had significantly higher SA levels in the SF as compared with patients with KL grade 2 (P<0.01). The SA levels in the SF of the knee OA patients positively correlated with the KL grades (r=0.353; P<0.01). However, there was no significant correlation identified between serum SA levels and KL grade. Therefore, SA levels in the SF positively correlated with the radiographic severity of OA, thus, SA levels in the SF may serve as a biomarker for the progression of OA.

## Introduction

Osteoarthritis (OA), a chronic degenerative joint disease, causes pain, stiffness, reduced motion, swelling, crepitus and disability. The disease is characterized by the progressive destruction of articular cartilage with joint-space narrowing, osteophyte formation, subchondral sclerosis and synovitis (1). A combination of risk factors, including aging, obesity, female gender, smoking, genetics, joint injury, mechanical and metabolic factors, have been shown to be involved in the pathophysiology of OA (2). However, the etiology and pathogenesis of OA remain poorly understood. Inflammation has been implicated in the pathogenesis of OA (3). Inflammatory markers, including interleukin-6, tumor necrosis factor-α (4) and C-reactive protein (5), in the serum and synovial fluid (SF) of OA patients have been reported to correlate with the radiographic severity of knee or hip OA.

Sialic acid (SA) is the terminal component at the non-reducing end of carbohydrate chains of glycoproteins and glycolipids (6). SA plays an important role in cell protection, fertilization, differentiation, adhesion, immunology and tumor development (7). Plasma SA is traditionally utilized as one of the biomarkers for the acute-phase response (8). Increased SA concentrations have been reported during inflammatory processes, possibly resulting from the elevated levels of richly sialylated acute-phase glycoproteins (9). In addition, elevated levels of SA have been reported to be associated with an increased risk of renal disease, diabetes, ovarian cancer and a variety of central nervous system disorders (10–12). A previous study demonstrated that patients with OA exhibited significantly elevated levels of serum SA when compared with healthy controls (13). Thus, it was hypothesized that SA may be involved in the mechanism underlying OA, and SA levels in the serum and SF may correlate with the severity of knee OA.

Although a number of studies (7,8,11,13) have investigated the differences in serum SA levels between OA patients and healthy controls, to the best of our knowledge, an investigation into the association between SA levels and disease severity in OA has not yet been performed. Therefore, the aim of the present study was to determine the correlation between SA concentrations in the serum and SF and radiographic disease severity in patients with knee OA in order to assess the role of SA in OA pathophysiology.

## Materials and methods

### Patients

A total of 234 patients diagnosed with knee OA, according to the criteria of the American College of Rheumatology (13), were enrolled in the present study. Patients that had acute or chronic inflammatory knee disease, rheumatoid arthritis (RA), systemic or autoimmune diseases or previous knee trauma were excluded from the study. The control group consisted of 160 healthy subjects with no clinical or radiological evidence of OA. Furthermore, the study included 20 patients that had suffered a knee injury or fracture, thus, knee SF samples were collected from these individuals. The study was approved by the Ethics Committee of the Beijing Charity Hospital (Beijing, China) and informed consent was provided by all the subjects.

### Radiographic assessment of OA

Disease severity assessments were performed using the Kellgren-Lawrence (KL) grading system (14). Subjects who had radiographic knee OA of KL grade ≥2 in at least one knee were defined as OA patients. Subjects who had KL grade 0 for both knees were defined as healthy controls. When the patients were affected in both knees, the grading of the worst affected knee was used for analysis.

### Laboratory methods

Venous blood samples were collected from all the subjects following overnight fasting. Prior to any OA treatment, SF was obtained from the OA patients that were receiving hyaluronic acid injection treatment for the first time. SA levels in the serum and SF samples were determined using Warren’s thiobarbituric acid assay (15).

### Statistical analysis

Data are presented as the mean ± SD or the median (interquartile range). Data normality was analyzed using the Kolmogorov-Smirnov test. The unpaired t-test, Mann-Whitney U test and χ^2^ test were utilized to analyze the differences in clinical characteristics between the knee OA patients and the healthy controls. The Kruskal-Wallis test was used to compare the differences in SA levels in the serum and SF of the knee OA patients with various KL grades. Statistical significance of the correlation between the SA levels in the serum and SF and disease severity was determined by Spearman’s coefficient and multinomial logistic regression analyses. As SA levels were not normally distributed, logarithmic transformed values were used for multiple linear regression analysis. All statistical analyses were performed using SPSS 13.0 for Windows (SPSS, Inc., Chicago, IL, USA) and P<0.05 was considered to indicate a statistically significant difference.

## Results

### Baseline clinical parameters

Baseline clinical parameters of the knee OA patients and healthy controls are shown in Table I. There were no significant differences in age, gender or body mass index (BMI) between the two groups.

### SA levels in the serum and SF

**Figure 1:**
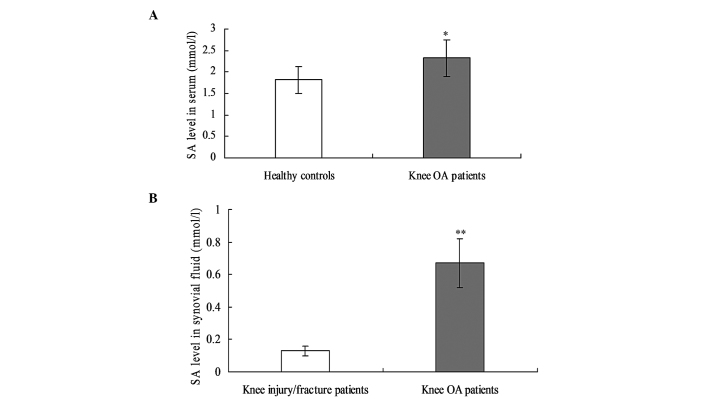
SA levels in the (A) serum and (B) SF of knee OA patients. ^*^P<0.05, vs. healthy controls; ^**^P<0.01, vs. knee injury/fracture patients. SF, synovial fluid; SA, sialic acid; OA, osteoarthritis.

Patients with knee OA exhibited significantly elevated serum SA levels as compared with the healthy controls (Table I; Fig. 1A; P<0.05). In addition, the SA levels in the SF increased significantly when compared with the knee injury or fracture patients without knee OA (Fig. 1B; P<0.01).

### SA levels in knee OA patients with various KL grades

**Figure 2:**
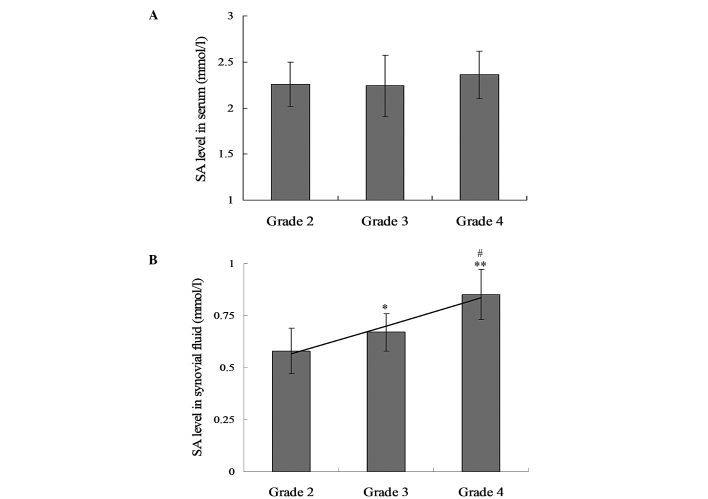
Association between SA levels in the (A) serum and (B) SF with various KL grades. ^*^P<0.05 and ^**^P<0.01, vs. grade 2 OA patients. ^#^P<0.05, vs. grade 2 OA patients. SF, synovial fluid; SA, sialic acid; OA, osteoarthritis; KL, Kellgren-Lawrence.

SA levels in the serum and SF of knee OA patients with various KL grades are shown in Table II. No statistically significant differences in the serum SA levels were identified between the patients with various KL grades (Fig. 2A; P=0.299). However, knee OA patients with KL grade 4 had significantly higher SA levels in the SF when compared with KL grade 2 (P<0.01) and 3 patients (P<0.05; Fig. 2B). In addition, higher SA levels in the SF were identified in knee OA patients with KL grade 3 as compared with KL grade 2 OA patients (Fig. 2B).

### Correlation between clinical parameters and KL grades

**Figure 3:**
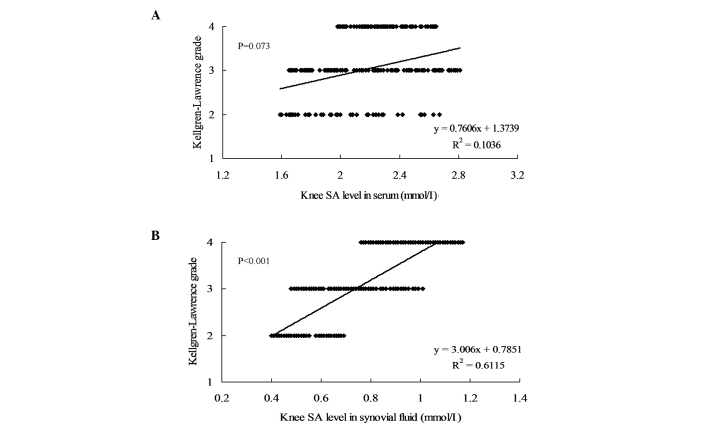
Correlation between KL grades and the SA level in the (A) serum and (B) SF of knee OA patients. SF, synovial fluid; SA, sialic acid; OA, osteoarthritus; KL, Kellgren-Lawrence.

Spearman’s correlation analysis indicated that the SA levels in the serum did not correlate with KL grades (Fig. 3; r=0.117; P=0.073). However, there was a significant positive correlation between SA levels in the SF and KL grades (Fig. 3; r=0.353; P<0.001).

Characteristics, including age, gender, BMI and SA levels in the serum and SF, were then analyzed using a multinomial logistic regression model. Multinomial logistic regression analysis demonstrated that SA levels in the SF positively correlated with KL grades (P<0.001). However, there was no significant correlation between the serum levels of SA and KL grades following multinomial logistic regression analysis ((Fig. 3; P=0.103).

## Discussion

The results of the present study indicate that patients with knee OA have significantly elevated levels of serum SA compared with healthy controls. In addition, SA levels in the SF positively correlated with KL grades. To the best of our knowledge, this is the first study to demonstrate that SA levels in the SF of knee OA patients correlate with the severity of OA.

OA is not considered to be a classical inflammatory disease mainly due to the absence of neutrophils in the SF and the lack of systemic manifestations of inflammation. However, OA is associated with symptoms of inflammation, including joint pain, swelling and stiffness, which result in functional impairment and disability (16). It is estimated that ~60% of joints affected by OA have synovial inflammation at the time of joint replacement (17). Numerous components of the complement cascade and other inflammatory mediators are reported to be elevated in the SF of OA patients (18). Serum SA concentration is elevated during the acute-phase reaction due to an increase in the serum concentration of SA-carrying acute-phase proteins, as well as a higher degree of sialylation of these proteins (19). It has been hypothesized that a single measurement of serum SA may be a useful estimate of the inflammatory status of an individual (20). The results of the present study indicated that serum SA levels were significantly elevated in patients with knee OA as compared with the healthy controls. In addition, SA concentrations in the SF exhibited a significant correlation with KL grades. These observations are consistent with those of a previous study, which demonstrated that OA patients had significantly higher serum SA levels than healthy controls (13). The current results indicate that SA may play an important role in the pathogenesis of OA, thus, SA levels may serve as a new biomarker for predicting the presence and progression of OA.

Serum SA levels have been shown to be elevated in patients with RA, as compared with healthy controls (13). Kosakai also reported that serum SA levels were moderately high in patients with stage 2 or 3 RA. Furthermore, SA levels in the SF were higher in cases of RA and within the normal range in cases of OA (21). The mechanisms of OA and RA are associated with inflammation. Thus, SA appears to contribute to the presence of OA and RA as an inflammatory factor.

There are several limitations of the present study. Firstly, this is a cross-sectional study performed with a relatively small sample size. Therefore, the observations should be validated by further longitudinal studies with a larger population sample. Secondly, SA levels in the SF from healthy controls were not assessed due to ethical concerns.

In conclusion, knee OA patients have significantly elevated serum SA levels as compared with healthy controls. In addition, SA levels in the SF positively correlate with the severity of knee OA. Therefore, SA levels in the SF may serve as a new biomarker, in addition to the traditional methods, for assessing the severity of knee OA.

## Figures and Tables

**Table I:** Characteristics of knee OA patients and healthy controls.

Characteristics	Knee OA patients (n=234)	Healthy controls (n=160)	P-value
Age, years	60.40±9.06	60.12±8.34	0.757
Gender, male/female, n	86/148	63/97	0.598
BMI, kg/m^2^	25.69±3.28	25.40±3.12	0.378
Serum SA, mmol/l	2.32 (1.74–2.72)	1.82 (1.49–2.31)	<0.05
SF SA, mmol/l	0.67 (0.48–0.96)		

OA, osteoarthritis; BMI, body mass index; SA, sialic acid; SF, synovial fluid.

**Table II:** SA levels in the serum and SF of knee OA patients with various KL grades.

SA, mmol/l	Grade 2 (n=64)	Grade 3 (n=98)	Grade 4 (n=72)	P-value
Serum	2.26 (1.59–2.67)	2.24 (1.65–2.81)	2.36 (1.98–2.65)	0.299
SF	0.58 (0.40–0.69)	0.67 (0.48–1.01)	0.85 (0.64–1.17)	<0.001

OA, osteoarthritis; SA, sialic acid; SF, synovial fluid; KL, Kellgren-Lawrence. P-values are for all three groups being compared.
